# Spatial organization of a soil cyanobacterium and its cyanosphere through GABA/Glu signaling to optimize mutualistic nitrogen fixation

**DOI:** 10.1093/ismejo/wrad029

**Published:** 2024-01-16

**Authors:** Corey Nelson, Pavani Dadi, Dhara D Shah, Ferran Garcia-Pichel

**Affiliations:** Center for Fundamental and Applied Microbiomics, Biodesign Institute, Arizona State University, Tempe, AZ 85287, United States; School of Life Sciences, Arizona State University, Tempe, AZ 85287, United States; Instituto Multidisciplinar Para Estudios del Medio “Ramon Margalef”, Universidad de Alicante, San Vicente del Raspeig 03690, Spain; Center for Fundamental and Applied Microbiomics, Biodesign Institute, Arizona State University, Tempe, AZ 85287, United States; School of Life Sciences, Arizona State University, Tempe, AZ 85287, United States; Center for Fundamental and Applied Microbiomics, Biodesign Institute, Arizona State University, Tempe, AZ 85287, United States; School of Mathematical and Natural Sciences, Arizona State University, Glendale, AZ 85306, United States; Center for Fundamental and Applied Microbiomics, Biodesign Institute, Arizona State University, Tempe, AZ 85287, United States; School of Life Sciences, Arizona State University, Tempe, AZ 85287, United States

**Keywords:** biocrusts, biological soil crusts, cyanobacteria, aggregation, motility, GABA, glutamate, microbiome

## Abstract

Soil biocrusts are characterized by the spatial self-organization of resident microbial populations at small scales. The cyanobacterium *Microcoleus vaginatus*, a prominent primary producer and pioneer biocrust former, relies on a mutualistic carbon (C) for nitrogen (N) exchange with its heterotrophic cyanosphere microbiome, a mutualism that may be optimized through the ability of the cyanobacterium to aggregate into bundles of trichomes. Testing both environmental populations and representative isolates, we show that the proximity of mutualistic diazotroph populations results in *M. vaginatus* bundle formation orchestrated through chemophobic and chemokinetic responses to gamma-aminobutyric acid (GABA) /glutamate (Glu) signals. The signaling system is characterized by: a high GABA sensitivity (nM range) and low Glu sensitivity (μM to mM), the fact that GABA and Glu are produced by the cyanobacterium as an autoinduction response to N deficiency, and by the presence of interspecific signaling by heterotrophs in response to C limitation. Further, it crucially switches from a positive to a negative feedback loop with increasing GABA concentration, thus setting maximal bundle sizes. The unprecedented use of GABA/Glu as an intra- and interspecific signal in the spatial organization of microbiomes highlights the pair as truly universal infochemicals.

## Introduction

Biological soil crusts (or biocrusts) are photosynthetic communities that play major roles in arid land soil stability and fertility, contributing significantly to the global C and N cycles [1-3]. *Microcoleus vaginatus*, a motile filamentous cyanobacterium, is the predominant pioneer of many biocrusts (see [4, 5] for reviews) and possibly the most abundant terrestrial cyanobacterium globally [6]. One of its defining traits is the ability to aggregate into bundles of trichomes held within a common sheath through dynamic motility responses [7] that help it attain macroscopic size and initially stabilize unconsolidated soils [7, 8]. While *M. vaginatus* cannot fix nitrogen, it does colonize N-limited bare soils due to its ability to spatially arrange other soil bacteria around it, repelling or avoiding competing cyanobacteria by reacting to their exudates [9] and attracting a mutualistic “cyanosphere microbiome” that trades newly fixed nitrogen in exchange for its photosynthates [10-14]. Further, bundling seems to be an integral part of the mechanism for mutualistic interaction [11], as it is enhanced in culture by N limitation and by the presence of specific cyanosphere mutualists. But the mechanism by which these mutualistic partners find each other in the soil and optimize their spatial architecture is currently unknown. Theoretically, populations of cyanobacteria may need to remain as bundles close to populations of appropriate diazotrophs that remain sessile on the bundle’s common sheath in order to optimize C for N exchanges. As C for N symbioses often involve exchanges of nitrogenous compounds (such as ammonium or amino acids) via diffusion and active transport [15-17], we hypothesized that such compounds could play a secondary role in interspecies signaling to guide interactions, as happens in other cyanobacteria–heterotroph associations [18, 19].

To understand factors leading to bundle formation as a driver of biocrust microbiome self-organization, we investigated the effects of N source, nutrient status, and microbial interactions on the motile and bundling behavior of *M. vaginatus*, using both pedigreed, cultivated representative strains and naturally existing biocrust populations. This led us to uncover the signaling role of the gamma-aminobutyric acid/glutamate (GABA/Glu) system behind bundle formation and spatial organization of the biocrust microbiome.

## Materials and methods

### Culturing and natural biocrust sourcing

*Microcoleus vaginatus* (PCC 9802), an axenic cyanobacterial strain isolated from biocrusts in the US southwest and available through the Pasteur Culture Collection of Cyanobacteria (Paris, France), was maintained in 250-mL vented suspension culture flasks (Greiner Bio-One, Kremsmünster, Austria) in 50% BG11 medium [20] at 23°C, 18–20 μE m^−2^ s^−1^ of white light illumination and 14 h light/10 dark cycle. To impose N-limitation, biomass was centrifugally washed (×3, 8000 rpm, 8 min) in N-free 50% BG11_0_ medium and incubated for 14 days. The diazo-heterotrophic strains (*Bacillus* sp. O64, *Arthrobacter* sp. O80, and *Massilia* sp. METH4), previously isolated from the cyanosphere of *M. vaginatus* [11] were maintained on Burks +1% gellan gum medium [21] in the dark. Biocrust used as a source of natural populations or for direct experimentation were sampled using 15-cm-diameter Petri dishes [22] from the Jornada Basin LTER, NM (Chihuahuan Desert) or from Mesa, AZ (Sonoran Desert). Biocrusts were air dried and maintained inactive at 15% RH in darkness until experimentation. Biocrust chl *a* concentration, a proxy for photosynthetic biomass, was determined spectrophotometrically as previously described [23-25].

### Growth of *M. vaginatus* on different N-sources

N-starved biomass was inoculated at 0.24 ± 0.04 mg chl *a* L^−1^ on 12-well plates containing 2 mL 50% BG11_0_ supplemented with 10 mM (final) of different potential nitrogen sources (*n* = 4 wells), unless otherwise stated. N-sources were nitrate, ammonium, urea, and amino acids representing all amino acid side groups (alanine, arginine, aspartate, cysteine, GABA, glutamine, glutamate, glycine, methionine, serine, tryptophan; Sigma-Aldrich, St. Louis, USA). All media were adjusted to pH 7 after the addition of amino acids or inorganic N, and NaCl was added to maintain equivalent ionic strength across treatments and controls. 100 μL homogenized aliquots from each well were taken initially and after 6 days, added to 900 μL of acetone in 2-mL microcentrifuge tubes containing 0.25 g of 0.5-mm zirconium beads, beat for 2 min at 30 s intervals, and then extracted in the dark for 24 h at 4°C. Chl *a* was determined from absorbance and absorption coefficients at 663 nm.

### Castenholz motility assays

For “Castenholz Motility Assays” [26-28], *M. vaginatus* PCC 9802 suspensions were inoculated into 12-well plates containing 1 mL of appropriate liquid media and allowed to acclimate under standard culture conditions for 24 h before measurements were taken. The working volume of wells within the 12- and 24-well plates used for experimentation were 3 mL and 1 mL, respectively. All growth media were adjusted to pH 7 after the addition of amino acids or inorganic N and supplemented with NaCl to maintain equivalent ionic strength across treatments, when appropriate. Cultures were then mixed to homogeneity by repeated in-and-out pipetting, photographed, allowed to entangle for 60 min of contraction, and photographed again. Responses were quantified by image analyses in ImageJ [29] as a percent change in areal cover of cyanobacterial biomass from dispersed to contracted states. Significance was tested with a one-way ANOVA using R [30], after arcsine square root transformation.

### Isolation and cleansing of environmental bundles

To obtain natural bundles, biocrusts were wetted with sterile RO water for 1 h. Thereafter, individual cyanobacterial bundles were pulled under the dissecting scope using Watchmaker’s forceps, and either used directly or cleansed by dragging through sterile 2% agar plates to various degrees as needed for experiments and as previously video-documented [31]. Repeated passages causes progressively larger losses of soil and heterotrophic populations attached to the bundle by simple sheer. In experiments for production of GABA and Glu, we conducted at least eight passes.

### Determination of trichome motility responses

Trichome motility responses were measured on cultured material or in natural populations excised from biocrusts. Identification of the cyanobacteria in individual bundles as *M. vaginatus* was by microscopic observation. Once taxonomically identified, individual bundles were placed in 24-well plates containing 0.5 mL liquid medium under appropriate experimental treatments, and then, gliding speeds and direction reversal frequency of individual trichomes (*n* = 12 for each) were measured under the compound microscope visually using an ocular micrometer and a stopwatch. Differences were tested for significance with a one-way ANOVA using R [30].

### Bundle stability assays

Bundles excised from natural crusts and taxonomically identified were transferred to wells of 24-well plates containing 0.5 mL of 50% BG11_0_ + 2% agar and the appropriate additions for the treatment at hand (*n* = 12 for each), initially photographed under the dissecting scope, incubated for 24 h in standard culture conditions, and photographed again. Images were analyzed in ImageJ [29] to determine the area occupied by trichomes spreading from the bundles, as well as the initial cross-sectional area of the bundle, from which an initial bundle volume of revolution was calculated. Stability was gauged as the final trichome spread area divided by the initial bundle volume. Differences in stability were tested with a one-way ANOVA using R [30]. For experiments requiring glutamate decarboxylase (GAD), a 10 μM purified GAD solution in 50% BG11_0_ was added. Heterologously expressed GAD from *Bacteroides fragilis* was purified in our laboratory. To visualize motile behavior *in situ*, intact biocrusts were fragmented into cm-sized pieces, distributed into six-well plates, supplemented with 1 mL of 50% BG11_0_ supplemented as necessary (*n* = 3), and incubated under standard culture conditions for 24 h. Photographs were taken initially and after 24 h for qualitative comparisons.

### Determination of GABA and glutamate

We analyzed GABA and Glu concentrations in the biocrust pore water (soil solution) and in growth medium of cultures toward the end of exponential growth. For biocrusts, 10 g samples were placed on a polycarbonate filter in a Büchner funnel, saturated with 10 mL of sterilized RO water, incubated for 1 h, and then flushed with 35 mL of sterile RO water, which was collected through vacuum filtration (*n* = 11). For heterotrophic cultures, 4-day-old spent medium was collected after centrifugation, and the pellet, which was cohesive, was weighed to determine biomass (*n* = 4) after blotting over tissue paper to remove excess water. For cyanobacteria cultures, biomass was determined after 14 days through chl *a* concentrations [36] (*n* = 4) and converted to wet biomass using a chl *a* content of 1% of DW or 0.2% of wet weight [32]. After collection, pore water or spent media were filter-sterilized and stored at 4°C for less than 48 h before filtration through 5 KDa MWCO 500-μL spin filters (Cytiva, Vivaspin) at 6000 g for 30 min and then stored at −20°C until analysis. GABA was measured spectrophotometrically by a coupled enzyme assay with a GABase mixture (Sigma) where GABA is converted to succinic semialdehyde and then to succinate with concurrent production of NADPH, whose absorbance can be measured at 340 nm [33, 34] and converted to concentrations using an extinction coefficient of 6220 M^−1^ cm^−1^, and where [NADPH] = [GABA]. Reactions contained 25 μL of the sample and 75 μL of GABase assay mix (10 mM ßME, 2 mM α-ketoglutarate, 600 μM NADP+, 30 μg (0.015 U/mL) [35] of GABase in 50 mM Tris–HCl, pH 8.6) and were incubated at room temperature for 90 min. For glutamate, samples were incubated overnight at 37°C with 5 μM of *B. fragilis* glutamate decarboxylase (GAD), heterologously expressed, and purified in our laboratory, in 50 mM sodium acetate, pH 4.7. GAD converts glutamate to GABA [33], which was measured by the GABase assay above. Sterile RO water or the appropriate uninoculated medium was used as controls. Initial GABA concentrations in samples without GAD addition were used as controls. Glu concentrations were calculated as: [Glu] = [GABA] with GAD – [GABA] without GAD.

## Results

### Effects of cyanosphere population size on bundle stability

**Figure 1:**
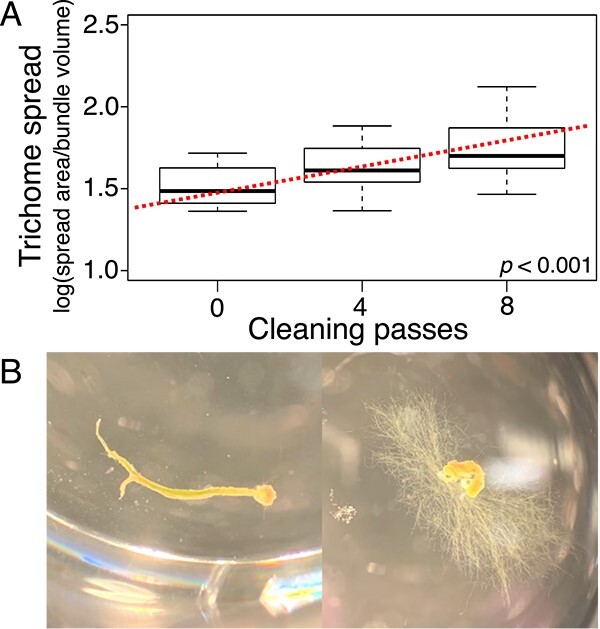
Effect of cyanosphere population size on bundle stability in natural bundles of *M. vaginatus*. (A) Stability, gauged as the inverse of the relative spread of *M. vaginatus* trichomes (*n* = 16) during a 24-h incubation, is plotted against the progressive removal of cyanosphere community from bundle sheaths (cleaning) by passage through agar. The slope of a linear regression (dotted line) is significantly different from 0 (ANOVA, *P* < 0.001). (B) Photographs showing a typical unstable, spreading bundle (right) and a stable, nonspreading bundle (left) in this type of assay.

To test if cyanosphere populations help keep *M. vaginatus* trichomes within bundles, we excised bundles directly from biocrusts and subjected these natural populations to increasingly intense physical cleansing to remove heterotrophs attached to the bundle sheaths. We then incubated cleansed bundles on solid media under N limitation, which promotes bundle conformation [11], for 24 h and quantified the relative proportion of trichomes that had left the original bundle using the bundle stability assay (BSA; Fig. 1). An inverse relationship between level of cleansing and bundle stability was evident and statistically significant (ANOVA, *P* < .01). Thus, the migration of trichomes away from bundles was positively correlated to a reduction of cyanosphere populations, which is consistent with the hypothesis that sheath-resident populations produce a diffusible cue that promotes bundle conformations in *M. vaginatus.*

### Nitrogen source effects on growth and aggregation of *M. vaginatus* PCC 9802

**Figure 2:**
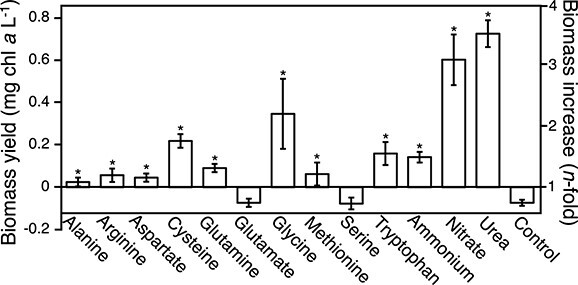
Biomass yield of *M. vaginatus* PCC 9802 with different potential N sources. Cultures were grown in N-free minimal medium after supplementation with 10 mM (final) of different compounds for 6 d (*n* = 4). Controls were given no supplement. Media was buffered at pH = 7. Asterisk denotes a (*P* < .05) significant difference in biomass yield from control.

To establish potential nitrogenous metabolites that may support the C for N mutualism between *M. vaginatus* and its cyanosphere, we grew axenic *M. vaginatus* in nitrogen-free medium with equimolar additions of either inorganic compounds or amino acids as a sole source of N (Fig. 2). *Microcoleus vaginatus* grew best on nitrate and urea but could also grow well on ammonium, cysteine, glycine, and tryptophan, and less efficiently on alanine, arginine, and aspartate. Biomass yields on glutamate (Glu) and serine were no different from those in N-free controls (ANOVA, *P* = 1 and .43, respectively), indicating that they could neither serve as a N source for growth nor likely support the N transfer from heterotrophs to phototroph.

**Figure 3:**
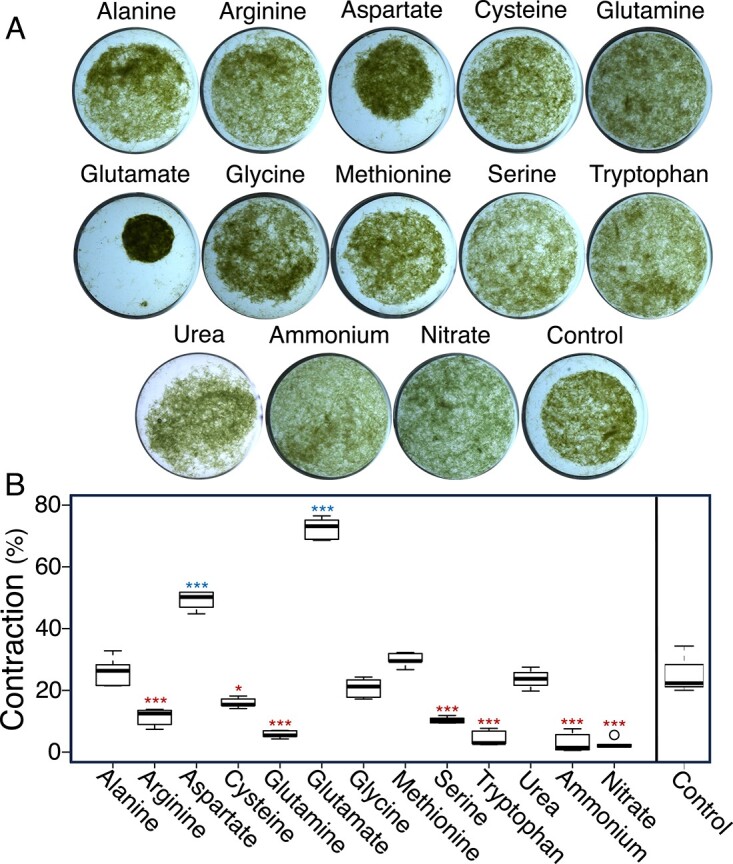
Effects of potential N sources on motility of *M. vaginatus* PCC 9802. Levels of contraction after 1 h of N-starved culture suspensions preacclimated for 24 h to 10 mM of different potential nitrogen sources added during acclimation. (A) Qualitative aspects of the assay. (B) Quantitative responses (*n* = 6), given as percent areal change from initial. Asterisks denotes either a significant increase (blue) or decrease (red) from control (^*^ stands for *P* < .05, ^*^^*^^*^ stands for *P* < .001) in ANOVA tests with post hoc *t*-tests corrected for multiple comparisons.

To test if any of these compounds guide *M. vaginatus* motility responses, we used Castenholz’ clumping assay (see Materials and Methods and Supplementary Video 1) based on the quantitation of macroscopically visible motility-driven entanglement of trichomes in a liquid suspension. The assay represents an integrative assessment of several types of motility responses that can contribute to apparent aggregation. Aggregation dynamics were clearly influenced by the available N source (Fig. 3), with Glu having the strongest effect, followed by aspartate, both leading to significantly stronger aggregation than N-free controls (ANOVA, *P* < .001, for both). Other N sources either made no difference (alanine, glycine, methionine, urea) or significantly diminished aggregation over N-free controls (ANOVA, *P* < .05 for all remaining; Fig. 3B). Glu was the only compound tested that induced aggregation but could not be used as N source for growth, suggesting a specific role as a signaling molecule to motility behavior. The effect of aspartate, though significant, was not further studied here as aspartate could be used by *M. vaginatus* as a N source for growth. While aspartate is a common signaling compound in bacterial chemotaxis [36], the observed effect of aspartate could be potentially ascribed to a lack of specificity in transporters or signal processing proteins, given its molecular similarity with Glu. Responsivity to Glu after an initial pulse of exposure slowly faded away within 4–5 h of its removal (Supplementary Fig. 1), indicating a tight regulation of sensitivity.

### Motility responses of *M. vaginatus* to glutamate

**Figure 4:**
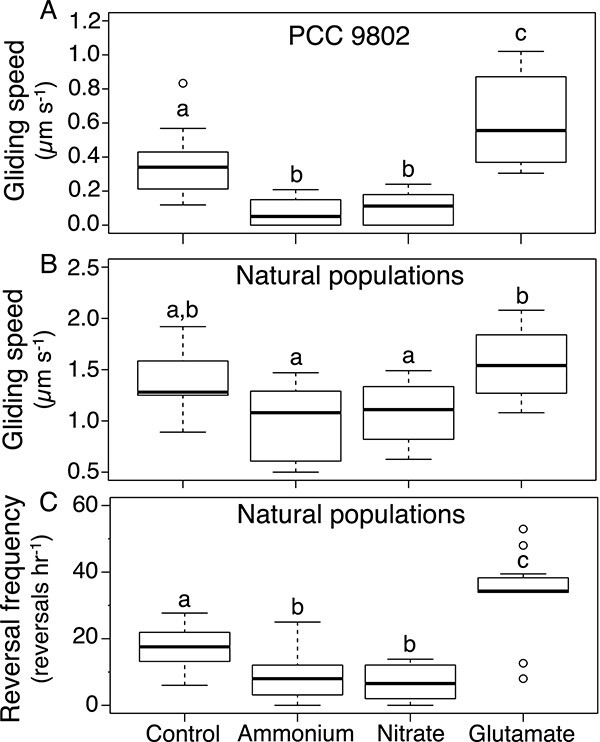
Chemokinetic and gliding reversal responses of representative isolate and natural populations of *M. vaginatus* to different nitrogen sources. (A) Chemokinetic responses of N-starved strain PCC 9802. (B) Chemokinetic responses of natural populations. (C) Gliding reversal response of natural populations. All samples (*n* = 12 per treatment) were incubated in minimal medium supplemented with 10 mM of each compound for 24 h. Controls had no N source. Statistical difference set as *P* < .01 in ANOVA with post hoc *t*-tests corrected for multiple comparisons, and different letters (a, b, c) denote significance differences among treatments.

To determine the basis for the macroscopic aggregation, we examined behavioral responses of *M. vaginatus* at the organismal (trichome) level. Gliding trichome speeds of N-starved strain PCC 9802 showed marked differences upon exposure to different N sources (Fig. 4A). Glu elicited a positive chemokinetic response, with speeds almost doubling those of the N-free control (ANOVA, *P* < .01). Conversely, exposure to either ammonium or nitrate slowed trichomes to around one-third of controls (ANOVA, *P* < .01 for both, and nondifferent between them *P* = .97). While the motility responses of gliding cyanobacteria typically include modulations in the frequency of gliding direction reversal, model strain PCC 9802 apparently lost this capacity to any measurable extent through decades of laboratory cultivation. To compensate for this, and to investigate the universality of our culture-based findings, we extended assays to environmental bundles excised from biocrusts by micromanipulation. After 24-h incubation, environmental *M. vaginatus* trichomes also showed differing motility responses to various N sources. Gliding speeds of environmental *M. vaginatus* were typically higher than those measured in strain PCC 9802, but the differential responses were similar (Fig. 4B). Compared to the N-free controls, trichomes exposed to Glu exhibited a slight, albeit nonsignificant (ANOVA, *P* = .26) positive chemokinesis, while trichomes exposed to ammonium and nitrate exhibited lower gliding speeds (also nonsignificant compared to controls; ANOVA, *P* = .48 and *P* = .60, respectively). However, differences between Glu and either ammonium or nitrate exposures were significant (ANOVA, *P* < .05 for both). Additionally, gliding reversal frequency was also differentially affected (Fig. 4C). Glu doubled reversal frequency over controls (ANOVA, *P* < .001), whereas ammonium or nitrate halved the frequency, respectively (ANOVA, *P* < .05 for both). A different type of motility response (true chemotaxis, an ability to move in the direction of a gradient of attractant) can sometimes be ascribed to cyanobacterial responses. In our case, while we could demonstrate PCC 9802’s ability to respond phototactically, and while this strain responds chemotactically to other chemical cues [9], it did not respond chemotactically to Glu (Supplementary Fig. 2).

### Glutamate as a cyanosphere match-maker

**Figure 5:**
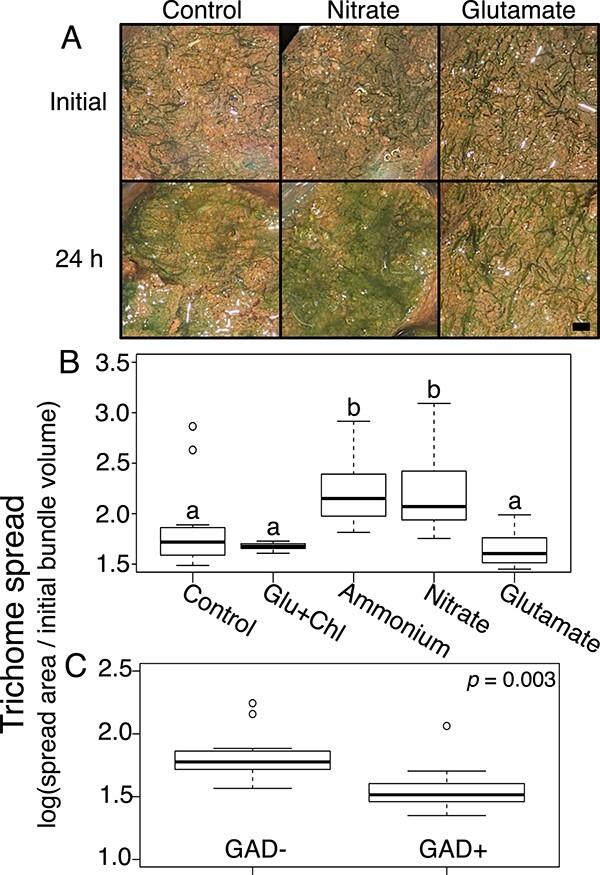
Effects of N source on bundle stability in natural populations of *M. vaginatus*. (A) Natural biocrust incubated for 24 h in minimal medium supplemented with either 10 mM of either nitrate or glutamate (*n* = 3)*.* Controls had no supplement. Shown are typical results from one experiment. Trichomes appear as discrete bundles or spread outside the bundles as greenish veils. Scale bar is 1 mm. (B) Degree of spread of *M. vaginatus* trichomes out of bundles (*n* = 12; see Fig. 1 for assay details) as a function of 10 mM N source. Statistical differences denoted by letters according to ANOVA post hoc *t*-test corrected for multiple comparisons (*P* < .05). (C) Degree of spread of *M. vaginatus* trichomes from bundle sheaths when incubated with (GAD+) or without (GAD−) glutamate decarboxylase in nitrogen-free minimal medium for 24 h (*n* = 12)*.* Incubation with GAD significantly increased bundle stability (*t*-test, *P* = .003).

One could predict from the previous results that exposure to Glu under N limitation will result in trichomes moving faster, but reversing much more often than in its absence. This will make the population stay in place, promoting trichome crowding within the bundle. Using the BSA (Fig. 5B), we could consistently show that field trichomes exposed to Glu tended to remain in or near the bundle, while those exposed to nitrate or ammonium tended to leave the bundle and spread out, compared to N-free controls (ANOVA, *P* < .05). We could further test this directly *in situ*, taking advantage of observations that, if maintained under wet conditions for extended periods, motile biocrust cyanobacteria tend to leave their sheath “tracks” and spread as single trichomes over the soil surface [37], creating a thin greenish veil. We thus monitored the distribution of surface trichomes between bundled and free trichome conformations after incubation with solutions containing Glu, nitrate, or no additions. Indeed, differences were marked: glutamate enhanced the proportion of in-bundle trichomes, whereas the presence of nitrate promoted their spread as single trichomes (Fig. 5A).

This suggested that the unknown cyanosphere signaling factor from previous experiments could indeed be glutamate (Fig. 1). To test this strictly, we attempted to interrupt an alleged Glu-based interspecies communication by artificially lowering the extracellular levels of Glu in active mutualisms. To do this, we incubated natural bundles in the presence of purified glutamate decarboxylase (EC 4.1.1.15; GAD), which decarboxylates glutamate to gamma-aminobutyric acid (GABA). Theoretically, incubations with excess enzyme lasting 24 h should have brought Glu well below the concentrations that elicits responses (see below). Our prediction was that lowered extracellular concentrations would impede signaling and lead to eventual loss of bundle organization. However, we observed the exact opposite result: bundles subject to GAD activity (GAD+) were more stable than controls (GAD-) (*t*-test, *P* = .003; Fig. 5C).

### Differential response of *M. vaginatus* to GABA and Glu

**Figure 6:**
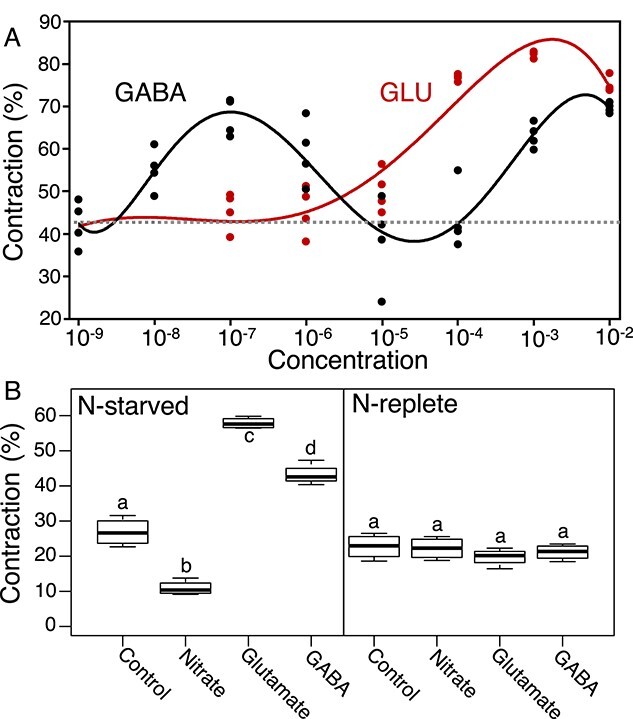
Concentration and N-status dependence of the aggregation response of *M. vaginatus* PCC 9802 for Glu and GABA. (A) Motility response assays as in Figure 2 (*n* = 4 per compound and concentration). Red and black trendlines represent average motility response to concentrations of Glu and GABA, respectively. Dotted line represents the average response of *M. vaginatus* in controls with no additions, ran concurrently. (B) Strain PCC 9802 preincubated in either N-replete or N-free medium for 14 days and then acclimated to 10 mM of different N sources for 1 day, before assaying for 1-h contraction levels (*n* = 6). Statistical differences as *P* < .01 in ANOVA with post hoc *t*-tests corrected for multiple comparisons denoted by different letters.

A possible explanation for the unexpected result above is that GABA itself can elicit responses more strongly than Glu, so that GAD activity would in fact enhance the response. We then characterized aggregation responses of N-limited *M. vaginatus* PCC 9802 to a wide concentration range of both Glu and GABA. For Glu, maximal responses were attained between 100 μM and 10 mM (Fig. 6A) and were barely detectable at 10 μM. GABA did indeed elicit swift aggregation as well, displaying a bimodal concentration dependence. A maximal response was attained at 100 nM GABA, with measurable responses down to 10 nM, and a second maximum detected around 10 mM (Fig. 6A). As expected (Fig. 6B), only N-starved *M. vaginatus* responded to Glu or GABA (ANOVA, *P* < .001 for either).

**Figure 7:**
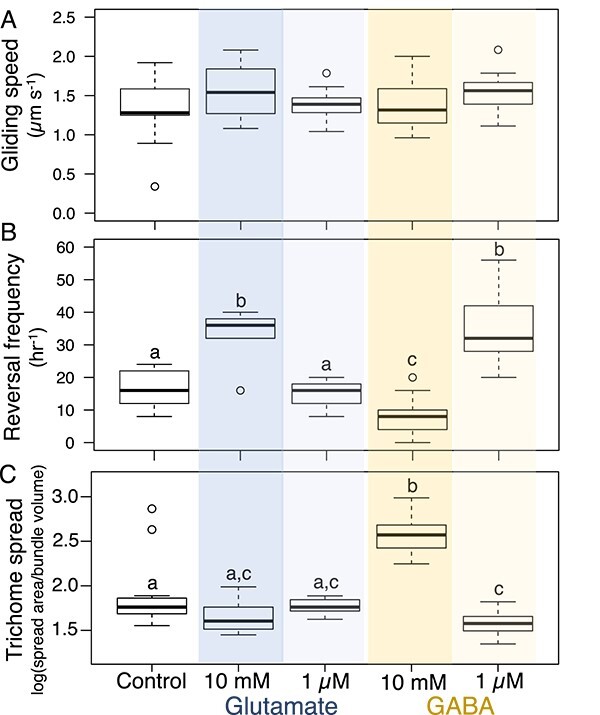
Comparative effects of GABA and Glu on motility responses of natural *M. vaginatus* populations. (A) Chemokinetic responses. (B) Gliding reversal response. (C) Degree of spread of *M. vaginatus* trichomes out of bundles as a function of GABA/Glu concentration. Single bundles were pulled from soil biocrust and incubated in minimal medium supplemented with different nitrogen sources for 24 h (*n* = 12 per treatment). Statistical differences are denoted by letters according to ANOVA post hoc *t*-test corrected for multiple comparisons (*P* < .05). Differences in gliding speed were not statistically significant. Assays are as described in Figures 1 and 4.

To determine the motility mechanisms leading to aggregation and to extend our culture-based findings to natural populations, we compared motility responses in environmental *M. vaginatus* at low (1 μM) and high (10 mM) concentrations of GABA and Glu (Fig. 7). Gliding speeds increased slightly with exposure to either 1 μM GABA or 10 mM Glu over the N-free controls, but they did not with exposure to either 1 μM Glu or 10 mM GABA. However, these differences were not significant (ANOVA, *P* > .65 for all; Fig. 7A). The effects on reversal rates were more marked. One micromolar GABA or 10 mM Glu doubled reversal frequency and were different from the control (ANOVA, *P* < .001) but were not significantly different from each other (ANOVA, *P* = .997). Predictably, 1 μM Glu produced no significant changes in reversal frequency over controls (ANOVA, *P* = .94). However, 10 mM GABA did not increase reversal rates, but rather, more than halved its frequency (ANOVA, *P* < .03; Fig. 7B).

**Figure 8:**
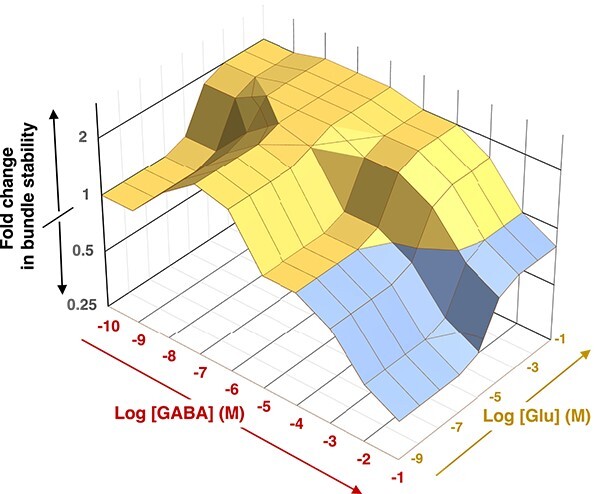
*Microcoleus vaginatus* bundle stability as a function of GABA and Glu concentrations. Relative bundle stability is measured as fold change from that in the absence of both compounds and is reconstructed combining the results in Figures 6 and 7. Yellow areas denote increased stability and blue areas decreased stability.

That GABA increased reversal frequency like Glu did, but at 1000-fold lower concentrations, suggests that such low concentrations of GABA would also be sufficient to induce increased bundle stability as was observed with Glu. Indeed, we found that to be the case: 1 μM GABA had a similar bundle stabilizing effect as 10 mM Glu (ANOVA, *P* = .97), significantly increasing stability over control or 1 μM Glu (ANOVA, *P* < .08 and .11, respectively; Fig. 7C). The bundle destabilizing effect of GABA at high concentration that one could have predicted from the halving of reversal frequency did indeed also materialize experimentally as significant differences in trichome spread between 10 mM GABA, controls, and all other treatments (Fig. 7C). This response divergence between GABA and Glu indicates that their sensing relies on somewhat differentiated mechanisms. Combining the information obtained from cultures (Fig. 6) and from environmental bundles (Fig. 7), it is possible to deduce the dependence of bundle stability as a function of Glu and GABA concentrations (Fig. 8).

### GABA/Glu production in bacterial cultures and natural communities

**Figure 9:**
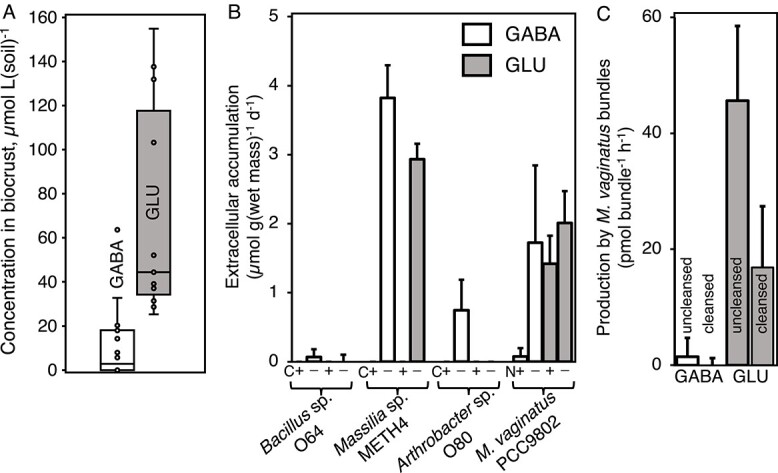
GABA and Glu production in cultures and natural communities. (A) Concentrations attained in natural biocrusts (*n* = 11)*.* (B) Biomass-specific extracellular accumulation per day in spent media of relevant isolates, as a function of nutritional status (*n* = 4) (C− starved for carbon and C+ carbon replete for isolates O64, METH4, and O80; N− starved for nitrogen and N+ nitrogen replete for isolate PCC 9802; error bars are two standard deviations of *n* > 3. (C) Production by environmental bundles with a full (uncleansed) or with diminished (cleansed) cyanosphere (*n* = 5 independent samples of 15–55 individual bundles each).

The responsivity to Glu and GABA (Fig. 8) highlights their potential as a signaling metabolite for *M. vaginatus* under N limitation. If true, GABA or Glu should be produced by at least some of the mutualistic partners, released to the exometabolome under appropriate environmental conditions, and accumulate in the environment to concentrations capable of inducing motility responses. To determine this, we measured GABA and Glu concentrations in natural biocrusts varying in origin, collection season, and level of development. While concentrations were quite variable among samples, both Glu and GABA were detectable. Mean Glu concentrations in biocrusts reached 71 ± 5 μmol L^−1^ of soil (*n* = 11; ranging from 25 to 138 μmol L^−1^ of soil) and GABA attained 11 ± 15 μmol L^−1^ of soil (*n* = 20; ranging from undetectable to 64 μmol L^−1^ of soil). The range of concentrations measured in natural biocrusts (Fig. 9A) does indeed make it possible for both compounds to play a role in motility responses, judging from the concentration ranges for GABA and Glu that induced motility responses in our experiments (Fig. 6).

To test for potential sources of GABA and Glu, we analyzed the spent growth medium of the mutualistic partners in culture (*M. vaginatus* PCC 9802 and three cyanosphere heterotrophs: *Bacillus* sp. O64, *Arthrobacter* sp. O80, and *Massilia* sp. METH4). Under N replete conditions, *M. vaginatus* itself produced barely detectable levels of GABA; around 0.08 μmol g^−1^ of biomass d^−1^, reaching extracellular concentrations around 2 μM. However, production was enhanced more than 20-fold under N-limitation; around 1.73 μmol g^−1^ of biomass d^−1^, reaching media concentrations around 52 μM (Fig. 9B). None of the cyanosphere heterotrophs produced detectable GABA under carbon-replete conditions, but all three produced some under C limitation (Fig. 9B). *Arthrobacter* sp. O80 produced 0.74 μmol GABA g^−1^ of biomass d^−1^ (reaching 3.8 ± 2.2 μM in the medium), *Massilia* sp. METH4 produced 3.82 μmol g^−1^ of biomass d^−1^ (reaching 17.2 ± 2.1 μM in the medium), and *Bacillus* sp. O64 produced 0.07 μmol g^−1^ of biomass d^−1^ (reaching 1.9 μM in the medium). Thus, not only members of the cyanosphere but also *M. vaginatus* itself are potential contributors to the GABA signaling pool when under conditions leading to mutualism (C or N limitation, respectively). Glutamate, by contrast, was only produced in measurable quantities by *M. vaginatus* PCC 9802 and *Massilia* sp. METH4*.* The cyanobacterium produced it under N-replete and N-free conditions, though production was significantly higher under N limitation (2.01 μmol g^−1^ of biomass d^−1^; *t*-test, *P* = .006) than under N-replete growth (1.42 μmol g^−1^ of biomass d^−1^), reaching concentrations 10–20 μM in the medium. In *Massilia* sp. METH4, Glu production (2.94 μmol g^−1^ of biomass d^−1^) occurred only under C limitation, with media concentrations around 13 μM. Again here, both cyanosphere members and *M. vaginatus* are potential contributors to the Glu signaling pool in a manner that is enhanced under conditions leading to mutualism.

In order to ascertain the relative role of heterotrophic vs. phototrophic mutualistic partners in driving signaling compound concentrations under natural conditions, we carried out an experiment in which environmental bundles of *M. vaginatus* were collected directly from soil, incubated in the light together with their surrounding cyanosphere bacterial population intact (i.e. uncleansed), or reduced (cleansed) to determine their production potential for Glu and GABA. In the uncleansed, mixed communities, we detected production of both Glu and GABA, Glu production exceeding that of GABA by about 30-fold (Fig. 9C). Cleansing of the cyanosphere resulted in significant decreases (*t*-test, *P* = .011; by about 2/3) of Glu production and also in a decrease in GABA to undetectable levels, although this decrease was not statistically significant (*t*-test, *P* = .37) (Fig. 9C) since we were operating close to the analytical limit of GABA detection of our assay.

## Discussion

### GABA/Glu as microbial signaling molecules

GABA and glutamate are important signaling molecules in animals, acting as potent neurotransmitters [38], as well as in plants where the role of GABA signaling has also been recently established [39, 22]. In some cases, plant GABA influences interactions between plants and associated pathogenic or mutualistic microbes [23, 29, 25], and some of the GABA-mediated control of microbial behavior described in the literature is exerted through interference with bacterial quorum sensing systems [41]. GABA and Glu are commonly synthesized and metabolized by a large variety of bacteria as a source of C or N, and many bacteria can chemotactically respond to them [27, 28], but there were until now no reports of either compound role in signaling among microbes. There is, however, much scientific interest in understanding microbial interactions with GABA and Glu as evidence suggests that the gut microbiome could influence human neurology through the gut–brain axis by their consumption or production [42]. The research presented here clearly speaks for the signaling role of the amino acid pair in mutualistic interactions within soil microbiomes in that (i) they directly elicit behavioral responses in the cyanobacterial partner, (ii) they are produced and excreted by various community members to concentrations sufficiently high to elicit these responses, and (iii) this signaling occurs in a manner regulated in response to their informational value, rather than nutritional value (Fig. 9). It is possible that GABA constitutes the biochemically true signaling compound at the receiving end and that the much weaker responses to Glu are the consequence of bleed-through from extracellular decarboxylation of Glu to GABA (enzymatic or spontaneous) in the soil or in culture. In the end, however, this is immaterial for the phenomenon at hand. Likewise, it is as yet unknown if the signaling role of the Glu/GABA pair we established here could be generalizable to microbiomes other than biocrusts, but the potential for interference between fully microbial and “trans-kingdom” signaling would make this worth investigating in animal or plant host–associated microbiomes.

### GABA/Glu as cyanobacterial autoinducers for N starvation

We demonstrate that both GABA and Glu have the characteristics of autoinducers in the motility responses of *M. vaginatus* to N starvation*.* Quorum-sensing, or autoinduction, is a form of intercellular, intraspecific signaling dependent on the density of the population and other factors that allow populations to behave in a coordinated fashion [43, 44]. It is widespread in bacteria and has been reported in some cyanobacteria [45]. Glu excretion is enhanced by N starvation and always leads to filament aggregation (bundle formation), but *M. vaginatus* is relatively insensitive to it. By contrast, it is extremely sensitive to GABA, down to nM concentrations, which is a typical threshold in known quorum-sensing autoinducers [46]. Among cyanobacteria, GABA biosynthesis relies on the conversion of glutamate through GAD [40], and GABA does accumulate to variable levels among strains intracellularly [47]. While it is plausible that other biocrust cyanobacteria could exude these compounds under similar conditions, we could not test this hypothesis as other axenic strains of relevant pioneer cyanobacteria do not exist in culture collections. However, we found no prior reports of GABA excretion in cyanobacteria nor about its role as autoinducer in any bacterium in the literature. Consistent with this notion, the GAD gene is detectable in multiple publicly available *M. vaginatus* genomes [48], and genes related to quorum sensing, such as acyl-homoserine lactone acylase homologs, are consistently found in its close genomic neighborhood.

### Mechanism of action for autoinduced, size-constrained bundle formation

Together, the phenomenology of *M. vaginatus* signal responses suggests how the system may work in a manner consistent with observations in nature and in culture. Under N-replete conditions, any effects of self-excreted Glu (Fig. 9C) will be denied through motility responses elicited by inorganic N, as seen in the preference for single-trichome conformation under nitrogen-replete conditions (Fig. 5). Consistently, *M. vaginatus* is rarely seen as single trichomes in nature, as most biocrusts are N-limited [39]. Under N-limitation, however, self-excreted GABA/Glu will start to accumulate around single trichomes, promoting the formation of bundles in a self-enhancing manner at random locations. Eventually, these bundles will grow to contain many trichomes, the local concentrations of self-produced GABA rising to its second, opposite activity peak, now counteracting the bundle-forming effects of Glu alone, and halting the process or resulting in active bundle deconstruction. The dual nature of the responses to Glu and GABA, thus enables a first-positive-then-negative feedback loop that will cause bundles to initially form and then reach an equilibrium at a certain maximal size.

### Role of the cyanosphere

It is clear that bundle formation based on mere autoinduction as discussed in the previous section has no bearing on an eventual improvement of the N-limitation condition. To account for this, one must include interactions with diazotrophic members of the bundle-associated cyanosphere [11]. Logically, this would be accomplished if *M. vaginatus* would form bundles preferentially where populations of heterotrophic mutualists exist and particularly where they exist “primed” for metabolic exchanges through C starvation. The cyanosphere depletion experiments presented direct evidence for this (Fig. 1), showing that the cyanosphere directly helps keep *M. vaginatus* in its bundled conformation. This can be traced back to the GABA/Glu mechanism by showing that the production of signals in environmental bundles was to a large extent ascribable to cyanosphere mutualists (Fig. 9C). This conclusion is further supported by the direct measurement of either GABA and/or Glu excretion in pedigreed heterotrophic diazotrophs in culture (Fig. 9B) and by the fact that heterotroph production is clearly dependent on a physiological C limitation of growth. The fact that C-limited bacteria excrete valuable amino acids can only be understood if this brings some fitness benefit in ending C limitation, which is consistent with the proven role of attracting *M. vaginatus* to form C-exuding bundles to their proximity. Logically, the combined effects of cyanosphere-produced Glu and GABA will attract trichomes only until the autoinducing cyanobacterial GABA slows further recruitment, likely so that the provision of N for the cyanobacteria is still sufficient. A second feedback loop is also likely at play when bundles become too large around heterotrophic populations: cyanobacterial C excretions could relieve the limitations status of the cyanosphere, thus decreasing Glu/GABA signaling locally and leading to bundle disassembly. In this manner, cyanobacterial bundles will develop preferentially where diazotrophic populations exist and attain sizes commensurate to the heterotroph population density. This represents a mechanistically simple means of optimizing the exchange of C for N spatially within the soil.

### The importance of chemokinesis

If the only beneficial outcome of responses to GABA/Glu was to stay close to a sheath-bound cyanosphere, it would principally suffice to stop gliding, rather than to glide faster and reverse more often concurrently, as we found (Fig. 8). But if all trichomes within a bundle (easily tens to hundreds) were to remain static, those in the bundle core would receive considerably less, if any, benefits than those in the periphery. By maintaining active gliding, trichomes glide against each other in their typical ropelike conformation, randomizing their relative positions in time (Supplementary Video 2), distributing resources more equally, while the negative chemophobic response ensures that the population as a whole remains in the neighborhood where resources are found.

## Conclusion

We provide evidence for the role of the Glu/GABA pair as interspecies signaling molecules responsible for spatial organization of biocrust microbiomes to optimize C for N mutualisms, and for their role as an autoinducer molecule in the regulation of cyanobacterial bundle formation and size under N limitation. Further investigation into the role of GABA/Glu as signaling compounds in other microbiomes may be of interest, given its role in animal and plants systems as well.

## Author contributions

Corey Nelson (research concept, manuscript writing, experimental design, data analyses, figure preparation, results discussion, review manuscriot). Ferran Garcia-Pichel (research concept, manuscript writing, experimental design, data analyses, figure preparation, review manuscript). Pavandi Dadi (perform experiments an analyses, discuss results, review manuscript). Dhara Shah (perform experimenta an analyses, discuss results, review manuscript).

## Conflicts of interest

The authors declare no conflict of interest.

## Funding

This work was supported in part by the Jornada Basin LTER Graduate Research Fellowship Program to C.N. (DEB 2025166) and by National Science Foundation (DEB 2129537) to F.G.-P.

## Data availability

The datasets generated and analyzed during the current study are available from the corresponding author on reasonable request.

## Supplementary Material

Supplementary_Information_wrad029

Supplementary_videos_wrad029

## References

[1] Beraldi-Campesi H , Hartnett HE, Anbar A, Gordon GW, Garcia-Pichel F. Effect of biological soil crusts on soil elemental concentrations: implications for biogeochemistry and as traceable biosignatures of ancient life on land. Geobiology 2009; 7: 348–59. 10.1111/j.1472-4669.2009.00204.x. 19573165

[2] Reynolds R , Belnap J, Reheis M, Lamothe P, Luiszer F. Aeolian dust in Colorado Plateau soils: nutrient inputs and recent change in source. Proc Natl Acad Sci 2001; 98: 7123–7. 10.1073/pnas.121094298. 11390965 PMC34633

[3] Sancho LG , Belnap J, Colesie C, Raggio J, Weber B. Carbon budgets of biological soil crusts at micro-, meso-, and global scales. In: Weber B, Büdel B, Belnap J (eds). Biological Soil Crusts: An Organizing Principle in Drylands. 2016. Springer International Publishing, Cham, pp. 287–304. 10.1007/978-3-319-30214-0_15.

[4] Garcia-Pichel F. Desert environments: biological soil crusts. In: Bitton G (ed.) Encyclopedia of Environmental Microbiology, Vol. 6. Set. Wiley-Interscience, New York, USA, 2003, 1019–23.

[5] Belnap J , Büdel B, Lange OL. Biological soil crusts: characteristics and distribution. In: Belnap J, Lange OL (eds.) Biological Soil Crust: Structure, Function, and Management. Berlin: Springer-Verlag, 2001, 3–30.

[6] Garcia-Pichel F. Cyanobacteria. In: Schaechter M (ed). Encyclopedia of Microbiology, 3rd ed. 2009. New York: Academic Press, pp. 107–24. 10.1016/B978-012373944-5.00250-9.

[7] Garcia-Pichel F , Wojciechowski MF. The evolution of a capacity to build supra-cellular ropes enabled filamentous cyanobacteria to colonize highly erodible substrates. PLoS On*e* 2009;4:11, e7801. 10.1371/journal.pone.0007801. 19924246 PMC2773439

[8] Belnap J , Gardner J. Soil microstructure in soils of the Colorado Plateau: the role of the cyanobacterium *Microcoleus vaginatus*. West North Am Nat 1993;53:40–7.

[9] Nelson C , Giraldo-Silva A, Warsop Thomas F, Garcia-Pichel F. Spatial self-segregation of pioneer cyanobacterial species drives microbiome organization in biocrusts. ISME Commun 2022; 2: 114–23. 10.1038/s43705-022-00199-0. 37938289 PMC9723579

[10] Couradeau E , Giraldo-Silva A, De Martini F, Garcia-Pichel F. Spatial segregation of the biological soil crust microbiome around its foundational cyanobacterium, *Microcoleus vaginatus*, and the formation of a nitrogen-fixing cyanosphere. Microbiome 2019; 7: 111–22. 10.1186/s40168-019-0661-2. 30944036 PMC6448292

[11] Nelson C , Giraldo-Silva A, Garcia-Pichel F. A symbiotic nutrient exchange within the cyanosphere microbiome of the biocrust cyanobacterium, Microcoleus vaginatus. ISME J 2021; 15: 282–92. 10.1038/s41396-020-00781-1. 32968213 PMC7853076

[12] Baran R , Brodie EL, Mayberry-Lewis J, Hummel E, Da Rocha UN, Chakraborty R, et al. Exometabolite niche partitioning among sympatric soil bacteria. Nat Commun 2015; 6: 8289. 10.1038/ncomms9289. 26392107 PMC4595634

[13] Baran R , Lau R, Bowen BP, Diamond S, Jose N, Garcia-Pichel F, Northen TR Extensive turnover of compatible solutes in cyanobacteria revealed by deuterium oxide (D2O) stable isotope probing. ACS Chem Biol 2017; 12: 674–81. 10.1021/acschembio.6b00890. 28068058

[14] Pepe-Ranney C , Koechli C, Potrafka R, Andam C, Eggleston E, Garcia-Pichel F, Buckley DH Non-cyanobacterial diazotrophs mediate dinitrogen fixation in biological soil crusts during early crust formation. ISME J 2016; 10: 287–98. 10.1038/ismej.2015.106. 26114889 PMC4737922

[15] Lodwig EM , Hosie AHF, Bourde A, Findlay K, Allaway D, Karunakaran R, et al. Amino-acid cycling drives nitrogen fixation in the legume–*Rhizobium* symbiosis. Nature 2003; 422: 722–6. 10.1038/nature01527. 12700763

[16] Patriarca EJ , Tatè R, Iaccarino M. Key role of bacterial NH4+ metabolism in *Rhizobium*-plant symbiosis. Microbiol Mol Biol Rev 2002; 66: 203–22. 10.1128/MMBR.66.2.203-222.2002. 12040124 PMC120787

[17] Newton WE . Nitrogen fixation in perspective. In: Pedrosa FO, Hungria MGY, Newton WE (eds). Nitrogen Fixation: From Molecules to Crop Productivity. 2000. Kluwer Academic Publishers, Dordrecht, pp. 3–8.

[18] Paerl HW , Gallucci KK. Role of chemotaxis in establishing a specific nitrogen-fixing cyanobacterial-bacterial association. Scienc*e* 1985; 227: 647–9. 10.1126/science.227.4687.647. 17781825

[19] Beliaev AS , Romine MF, Serres M, Bernstein HC, Linggi BE, Markillie LM, Isern NG, Chrisler WB, Kucek LA, Hill EA, Pinchuk GE, Bryant DA, Steven Wiley H, Fredrickson JK, Konopka A Inference of interactions in cyanobacterial-heterotrophic co-cultures via transcriptome sequencing. ISME J 2014; 8: 2243–55. 10.1038/ismej.2014.69. 24781900 PMC4992078

[20] Stanier RY , Kunisawa R, Mandel M, Cohen-Bazire G. Purification and properties of unicellular blue-green algae (order *Chroococcales*). Bacteriol Rev 1971; 35: 171–205. 10.1128/br.35.2.171-205.1971. 4998365 PMC378380

[21] Wilson PW , Knight SG. Experiments in Bacterial Physiology, 3rd edn. Minneapolis, Minnesota: Burgess, 1952, 53

[22] Büdel B , Darienko T, Deutschewitz K, Dojani S, Friedl T, Mohr KI, Salisch M, Reisser W, Weber B Southern African biological soil crusts are ubiquitous and highly diverse in drylands, being restricted by rainfall frequency. Microb Ecol 2009; 57: 229–47. 10.1007/s00248-008-9449-9. 18850242

[23] Giraldo-Silva A , Nelson C, Barger N, Garcia-Pichel F. Nursing biocrusts: isolation, cultivation and fitness test of indigenous cyanobacteria. Restor Ecol 2019; 27: 793–803. 10.1111/rec.12920.

[24] Sorochkina K , Velasco Ayuso S, Garcia-Pichel F. Establishing rates of lateral expansion of cyanobacterial biological soil crusts for optimal restoration. Plant Soil 2018; 429: 199–211. 10.1007/s11104-018-3695-5.

[25] Giraldo-Silva A , Nelson C, Penfold C, Barger NN, Garcia-Pichel F. Effect of preconditioning to the soil environment on the performance of 20 cyanobacterial strains used as inoculum for biocrust restoration. Restor Ecol 2020; 28: 187–93. 10.1111/rec.13048.

[26] Hooper DU , Johnson L. Nitrogen limitation in dryland ecosystems: responses to geographical and temporal variation in precipitation. Biogeochemistry 1999; 46: 247–93. 10.1007/BF01007582.

[27] Kanwal S , Incharoensakdi A. Characterization of glutamate decarboxylase from *Synechocystis* sp. PCC6803 and its role in nitrogen metabolism. Plant Physiol Biochem 2016;99:59–65. 10.1016/j.plaphy.2015.12.008 26730883

[28] Castenholz RW . Aggregation in a thermophilic *Oscillatoria*. Nature 1967; 215: 1285–6. 10.1038/2151285a0.

[29] Castenholz RW . The behavior of *Oscillatoria terebriformis* in hot springs. J Phycol 1968; 4: 132–9. 10.1111/j.1529-8817.1968.tb04687.x. 27067949

[30] Richardson LL , Castenholz RW. Chemokinetic motility responses of the cyanobacterium *Oscillatoria terebriformis*. Appl Environ Microbiol 1989; 55: 261–3. 10.1128/aem.55.1.261-263.1989. 16347828 PMC184091

[31] Schneider CA , Rasband WS, Eliceiri KW. NIH Image to ImageJ: 25 years of image analysis. Nat Methods 2012; 9: 671–5. 10.1038/nmeth.2089. 22930834 PMC5554542

[32] R Development Core Team . R: A Language and Environment for Statistical Computing. R Foundation for Statistical Computing, Vienna, Austria. http://www.R-project.org/. 2020.

[33] Garcia-Pichel F , Loza V, Marusenko Y, Mateo P, Potrafka RM. Temperature drives the continental-scale distribution of key microbes in topsoil communities. Science 2013; 340: 1574–7. 10.1126/science.1236404. 23812714

[34] Heredia-Velásquez AM , Giraldo-Silva A, Nelson C, Bethany J, Kut P, González-de-Salceda L, Garcia-Pichel F Dual use of solar power plants as biocrust nurseries for large-scale arid soil restoration. Nat Sustain 2023; 6: 955–64. 10.1038/s41893-023-01106-8.

[35] Cozzani I. Spectrophotometric assay of l-glutamic acid decarboxylase. Anal Biochem 1970; 33: 125–31. 10.1016/0003-2697(70)90446-X. 4391728

[36] Tramonti A , De Biase D, Giartosio A, Bossa F, John RA. The roles of His-167 and His-275 in the reaction catalyzed by glutamate decarboxylase from *Escherichia coli*. J Biol Chem 1998; 273: 1939–45. 10.1074/jbc.273.4.1939. 9442028

[37] Tsukatani T , Higuchi T, Matsumoto K. Enzyme-based microtiter plate assay for γ-aminobutyric acid: application to the screening of γ-aminobutyric acid-producing lactic acid bacteria. Anal Chim Acta 2005; 540: 293–7. 10.1016/j.aca.2005.03.056.

[38] Clarke S , Koshland DE. Membrane receptors for aspartate and serine in bacterial chemotaxis. J Biol Chem 1979; 254: 9695–702. 10.1016/S0021-9258(19)83572-X. 385590

[39] Pringault O , Garcia-Pichel F. Hydrotaxis of cyanobacteria in desert crusts. Microb Ecol 2004; 47: 366–73. 10.1007/s00248-002-0107-3. 14605777

[40] Petroff OAC . GABA and glutamate in the human brain. Neurosci 2002;8:562–73 10.1177/1073858402238515 12467378

[41] Chevrot R , Rosen R, Haudecoeur E, Cirou A, Shelp BJ, Ron E, Faure D GABA controls the level of quorum-sensing signal in *Agrobacterium tumefaciens*. Proc Natl Acad Sci 2006; 103: 7460–4. 10.1073/pnas.0600313103. 16645034 PMC1464361

[42] Strandwitz P. Neurotransmitter modulation by the gut microbiota. Brain Res 2018; 1693: 128–33. 10.1016/j.brainres.2018.03.015. 29903615 PMC6005194

[43] Shapiro JA . Thinking about bacterial populations as multicellular organisms. Annu Rev Microbiol 1998; 52: 81–104. 10.1146/annurev.micro.52.1.81. 9891794

[44] Miller MB , Bassler BL. Quorum sensing in bacteria. Annu Rev Microbiol 2001; 55: 165–99. 10.1146/annurev.micro.55.1.165. 11544353

[45] Sharif DI , Gallon J, Smith CJ, Dudley E. Quorum sensing in cyanobacteria: N-octanoyl-homoserine lactone release and response, by the epilithic colonial cyanobacterium *Gloeothece* PCC6909. ISME J 2008; 2: 1171–82. 10.1038/ismej.2008.68. 18633449

[46] Kaplan HB , Greenberg EP. Diffusion of autoinducer is involved in regulation of the *Vibrio fischeri* luminescence system. J Bacteriol 1985; 163: 1210–4. 10.1128/jb.163.3.1210-1214.1985. 3897188 PMC219261

[47] Shiels K , Murray P, Saha SK. Marine cyanobacteria as potential alternative source for GABA production. Bioresour Technol Reports 2019;8:100342. 10.1016/j.biteb.2019.100342

[48] Starkenburg SR , Reitenga KG, Freitas T, Johnson S, Chain PSG, Garcia-Piche F, et al. Genome of the cyanobacterium *Microcoleus vaginatus* FGP-2, a photosynthetic ecosystem engineer of arid land soil biocrusts worldwide. J Bacteriol 2011; 193: 4569–70. 10.1128/JB.05138-11. 21705610 PMC3165530

