# Supplementary material for: Spatial organization of a soil cyanobacterium and its cyanosphere through GABA/Glu signaling to optimize mutualistic nitrogen fixation
Source: ISME J. 2024 Jan 16;18(1):wrad029. doi: 10.1093/ismejo/wrad029 (PMC10881301; doi:10.1093/ismejo/wrad029)
Supplement: Supplementary_Information_wrad029 [file supplementary_information_wrad029.docx]

**Supplementary Information**

**Spatial organization of a soil cyanobacterium and its cyanosphere through GABA/Glu signaling to optimize mutualistic nitrogen fixation**

Corey Nelson, Pavani Dadi, Dhara D. Shah and Ferran Garcia-Pichel

**Supplementary Figures**

**Supplementary Figure 1. Loss of responsivity dynamics after removal of Glu.**

*M. vaginatus* PCC9802 was N-starved and then preadapted for 24 h to 10 mM Glu. At time 0 cultures were maintained exposed to Glu (+Glu) or transferred to Glu-free medium (- Glu). Control cultures never were exposed to Glu. Motility response assays as in Fig. 3, were run recurrently on n=3 cultures per condition.

**Supplementary Figure 2. Tactic responses of *M. vaginatus* PCC9802.**

Assays were done on agar solidified minimal medium in response to gradients in glutamate (chemotaxis) and light (phototaxis) after 3 d (*n* = 7) and assessed as percentage of area swept either towards or away from the source of glutamate or light, which were located to the right of biomass. *Asterisks* denotes significance *p*<0.001. Typical outcomes shown in figures. Scale bar is 2 mm.

**Supplementary Video 1. Castenholz motility assays.**

One-hour time lapse photography of the contraction of *M. vaginatus* PCC9802 liquid suspensions in 12-well plates (2.8 cm diam.) with minimal nitrogen free medium (right), the same medium supplemented with 10 mM glutamate (left).

**(SEE SEPARATE FILE)**

**Supplementary Video 2. Environmental *M. vaginatus* gliding within bundles.**

Single trichomes glide against each other in a rope-like arrangement while held in a shared polysaccharide sheath. Single trichomes are 4-6 µm wide.

**(SEE SEPARATE FILE)**
